# Housing Adequacy and Home Modification Implementation Among Households With Older Adults Living in Them

**DOI:** 10.1093/geroni/igaf122.1779

**Published:** 2025-12-31

**Authors:** Bernard Steinman, Sung-Jin Lee, Randall Cantrell, Denise McAllister, Gina Peek, Andrew Carswell

**Affiliations:** University of Wyoming, Laramie, Wyoming, United States; North Carolina Agricultural and Technical State University, Greensboro, North Carolina, United States; University of Florida, Gainesville, Florida, United States; Texas State University, San Marcos, Texas, United States; Oklahoma State University, Stillwater, Oklahoma, United States; University of Georgia, Athens, Georgia, United States

## Abstract

Given the significance of aging in place (AIP) for fulfilling preferences of older adults and guiding policy options to improve their safety and wellbeing, it is important to understand relationships between amenable factors that may facilitate aging at home. The quality of homes in which older adults live can be a facilitator or a barrier to AIP. Housing adequacy is a measure of physical housing quality, which includes characteristics such as whether units have hot/cold running water, flush toilets, or electrical problems. Home modification (HM) is one potential strategy to improve the physical quality of the home environment and to accommodate functional changes experienced by many older people. This study employed data from the American Housing Survey to examine the relationship between housing adequacy and implementation of HMs during a two-year period (2017 through 2019). We controlled covariates for household-level demographics (e.g., age of householder, disability) and housing characteristics (e.g., housing type, year built) within a logistic regression model. Results show that housing adequacy was not associated with HM implementation. Among household demographic variables, education level and presence of at least one person with a disability predicted implementation of HMs in 2019. Among housing variables, age of home structure, metropolitan status, and whether HMs had been implemented previously were statistical predictors of HMs in 2019. Findings provide insights into household-level associations with HM implementation and may be used to inform better understanding of precursors as to why older people may choose to adapt their homes for AIP.

